# The Vertebrate Trait Ontology: a controlled vocabulary for the annotation of trait data across species

**DOI:** 10.1186/2041-1480-4-13

**Published:** 2013-08-09

**Authors:** Carissa A Park, Susan M Bello, Cynthia L Smith, Zhi-Liang Hu, Diane H Munzenmaier, Rajni Nigam, Jennifer R Smith, Mary Shimoyama, Janan T Eppig, James M Reecy

**Affiliations:** 1 Department of Animal Science, Iowa State University, Ames, IA, USA; 2 Mouse Genome Informatics, The Jackson Laboratory, Bar Harbor, ME, USA; 3 Human and Molecular Genetics Center, Medical College of Wisconsin, Milwaukee, WI, USA; 4 Department of Physiology, Medical College of Wisconsin, Milwaukee, WI, USA; 5 Department of Surgery, Medical College of Wisconsin, Milwaukee, WI, USA

**Keywords:** Quantitative trait loci, Gene association, Trait ontology

## Abstract

**Background:**

The use of ontologies to standardize biological data and facilitate comparisons among datasets has steadily grown as the complexity and amount of available data have increased. Despite the numerous ontologies available, one area currently lacking a robust ontology is the description of vertebrate traits. A trait is defined as any measurable or observable characteristic pertaining to an organism or any of its substructures. While there are several ontologies to describe entities and processes in phenotypes, diseases, and clinical measurements, one has not been developed for vertebrate traits; the Vertebrate Trait Ontology (VT) was created to fill this void.

**Description:**

Significant inconsistencies in trait nomenclature exist in the literature, and additional difficulties arise when trait data are compared across species. The VT is a unified trait vocabulary created to aid in the transfer of data within and between species and to facilitate investigation of the genetic basis of traits. Trait information provides a valuable link between the measurements that are used to assess the trait, the phenotypes related to the traits, and the diseases associated with one or more phenotypes. Because multiple clinical and morphological measurements are often used to assess a single trait, and a single measurement can be used to assess multiple physiological processes, providing investigators with standardized annotations for trait data will allow them to investigate connections among these data types.

**Conclusions:**

The annotation of genomic data with ontology terms provides unique opportunities for data mining and analysis. Links between data in disparate databases can be identified and explored, a strategy that is particularly useful for cross-species comparisons or in situations involving inconsistent terminology. The VT provides a common basis for the description of traits in multiple vertebrate species. It is being used in the Rat Genome Database and Animal QTL Database for annotation of QTL data for rat, cattle, chicken, swine, sheep, and rainbow trout, and in the Mouse Phenome Database to annotate strain characterization data. In these databases, data are also cross-referenced to applicable terms from other ontologies, providing additional avenues for data mining and analysis. The ontology is available at http://bioportal.bioontology.org/ontologies/50138.

## Background

The use of ontologies (formal, standardized vocabularies identifying the relationships between terms related to a particular subject matter) to standardize biological data and facilitate comparisons among datasets and across organisms has steadily grown as the complexity and amount of data available for researchers to analyze have increased. The hierarchical structure of ontologies makes them both machine readable and meaningful to human users, which results in more intuitive query and data display tools for investigators.

One of the largest and most widely used biological ontologies is the Gene Ontology (GO), which consists of three distinct controlled vocabularies used to describe the molecular functions, biological processes, and cellular components associated with gene products [1]. Ontologies have also been created to describe phenotypes [2,3], anatomy [4-7], cell types [8], chemical compounds [9], and proteins [10]. New ontologies continue to be developed at a rapid pace as evidenced by the National Center for Biomedical Ontology (NCBO; [11]), where the number of ontologies has increased from 72 in early 2008 [12] to 339 in April 2013.

Despite the numerous ontologies available, one area currently lacking a robust ontology is the description of vertebrate traits. A trait can be defined as any measurable or observable characteristic pertaining to an organism or any of its substructures. A search of ontologies to address the trait domain shows that while there are several ontologies that represent entities and processes in phenotypes, diseases, and clinical measurements, there has not been one for vertebrate traits; the Vertebrate Trait Ontology (VT) was developed to fill this void. Impetus for this project came from multiple groups including the Rat Genome Database (RGD; [13]), Mouse Genome Informatics (MGI; [14]), and the Animal QTL Database (QTLdb; [15]), and it began as a way to standardize descriptions and definitions of quantitative trait loci (QTL) for cross-species comparisons and other analyses. In addition, the need to link various levels of data connected with physiological processes, phenotypes, and disease mechanisms was identified.

The concepts of “phenotype” and “trait” are closely aligned, to the extent that some might consider them synonymous. However, while several phenotype ontologies exist, including the Mammalian Phenotype (MP) Ontology [2], the Human Phenotype (HP) Ontology [3], and the Phenotypic Quality Ontology (PATO; [16]), there are fundamental differences between the content and/or structure of these ontologies and the VT which make them less than ideal for expressing trait data. Neither the MP nor the HP fulfills this need because both ontologies are designed to express phenotypic variation from a “normal” state. For instance, although the HP *mode of inheritance* branch includes unaltered phenotypes, the other two branches, *onset and clinical course* and *phenotypic abnormality*, clearly indicate a more or less anomalous state. Likewise, the MP was specifically developed as a means to define the abnormal changes caused by mutations. Traits, on the other hand, do not indicate an abnormal state or process.

PATO is constructed in such a way that it would be possible to use it to express the normal state or process, but it differs from the VT in that it was created to annotate phenotypes using a combinatorial approach, in which a phenotypic character is composed of an entity (e.g., limb) and a quality, or attribute (e.g., length). PATO requires entities to be drawn from other ontologies, such as those describing anatomy or cell types [16]. Phenotype composition can be done either during ontology creation (pre-composition) or at the time of annotation (post-composition). One ontology that is pre-composed using PATO is the Fission Yeast Phenotype Ontology (FYPO; [17]). An example of a group that performs post-composition using PATO is the Zebrafish Information Network (ZFIN; [18]). Although the post-compositional approach facilitates computational analysis, it increases complexity and decreases ease of use for human users [19]. It also impedes curation, because more time is required for a curator to consult multiple ontologies to construct a single trait term. In addition, it increases the potential for ambiguity, since a compound term could be created in many ways depending on which ontologies the component terms are selected from (e.g., one may generate the term *circulating sugars amount* as an alternative to *blood glucose amount*).

Disease ontologies such as the Human Disease Ontology [20], SNOMED Clinical Terms [21], and the International Classification of Diseases [22] are not appropriate to express traits because the disease state is, by definition, abnormal. In addition, multiple traits may be associated with a disease and vice versa. While the Clinical Measurement Ontology (CMO) [23] does represent measurable entities, it is designed to describe the actual measurements taken which result in a quantitative or qualitative result and not the trait that the measurement is used to assess.

Trait information provides a valuable link between the measurements that are used to assess the trait, the phenotypes related to the traits, and the diseases associated with one or more phenotypes. A trait, such as erythrocyte size, is distinct from phenotype (a description of the manifestation of the trait; e.g., increased erythrocyte size) and measurement (a quantification or assessment of the trait; e.g., mean corpuscular volume). Significant inconsistencies exist in the literature when it comes to trait nomenclature. Even within species, multiple terms may be used to refer to the same trait (e.g., subcutaneous fat depth, subcutaneous adipose thickness, backfat thickness, etc.). Complexity increases when attempts are made to compare traits across species. Because multiple clinical and morphological measurements are often used to assess a single trait, and a single measurement can be used to assess multiple physiological processes, providing investigators with standardized annotations for trait data will allow them to investigate connections among these different types of data. Therefore, the Vertebrate Trait Ontology was developed to describe the measurable or observable characteristics pertaining to the morphology, physiology, and development of vertebrate organisms. It is available for public browsing and download via BioPortal (http://bioportal.bioontology.org/ontologies/50138).

### Construction and content

The VT was originally developed as an outgrowth of naming conventions and trait vocabularies utilized to characterize QTL. Its intended purpose was to assist in the discovery of cross-species syntenic regions identified as being associated with the same or similar traits. Because experimental techniques can differ widely depending on organism, and because many QTL were originally named and annotated according to terms used by authors, this cross-comparison proved difficult for many researchers. While individual entities such as MGI, RGD, QTLdb, and the French National Institute for Agricultural Research (INRA) each created limited naming conventions and vocabularies to more or less standardize QTL data within their own databases, there was little commonality among the groups. In addition, naming and trait assignment included disease terms, abnormal phenotype terms, measurements, and method terms, causing additional confusion.

The Vertebrate Trait Ontology was designed to create consistency in annotation across species and to provide a navigational layer among data types. Capitalizing on previous development efforts, the Mammalian Phenotype Ontology [2] was used as a basis for the VT. All references to abnormalities were stripped out, leaving a foundation of potential traits while retaining the structure of the MP. Each of the remaining terms was then reviewed to determine if it represented a “true” trait or would be more properly placed in a different ontology. To be considered a “true” trait, the term had to 1) meet the stated definition of a trait, i.e., “any measurable or observable characteristic pertaining to an organism or any of its substructures”; 2) be named and defined in terms of the characteristic itself and not measurements assessing that characteristic; and 3) be phenotype neutral. For example, *water intake rate* (CMO:0000741) is placed in the Clinical Measurement Ontology and not the VT since it reflects a measurement of a *drinking behavior trait* (VT:0001422). Likewise, while exencephaly (extrusion of the brain through the cranium) is an observable characteristic, it is not phenotype neutral, since it describes a particular type of head morphology. Within the VT, this observation would be annotated to the term *head morphology trait* (VT:0000432). Many terms were removed from the VT during this process. This left a skeletal set of higher level trait terms that continue to be expanded upon as the VT is used. The initial phase of expansion focused on addition of terms to cover existing QTL in the QTLdb and RGD, as well as terms requested by INRA. In addition, terms were added to include strain characterization traits needed for annotation at the Mouse Phenome Database (MPD; [24,25]).

**Figure 1:**
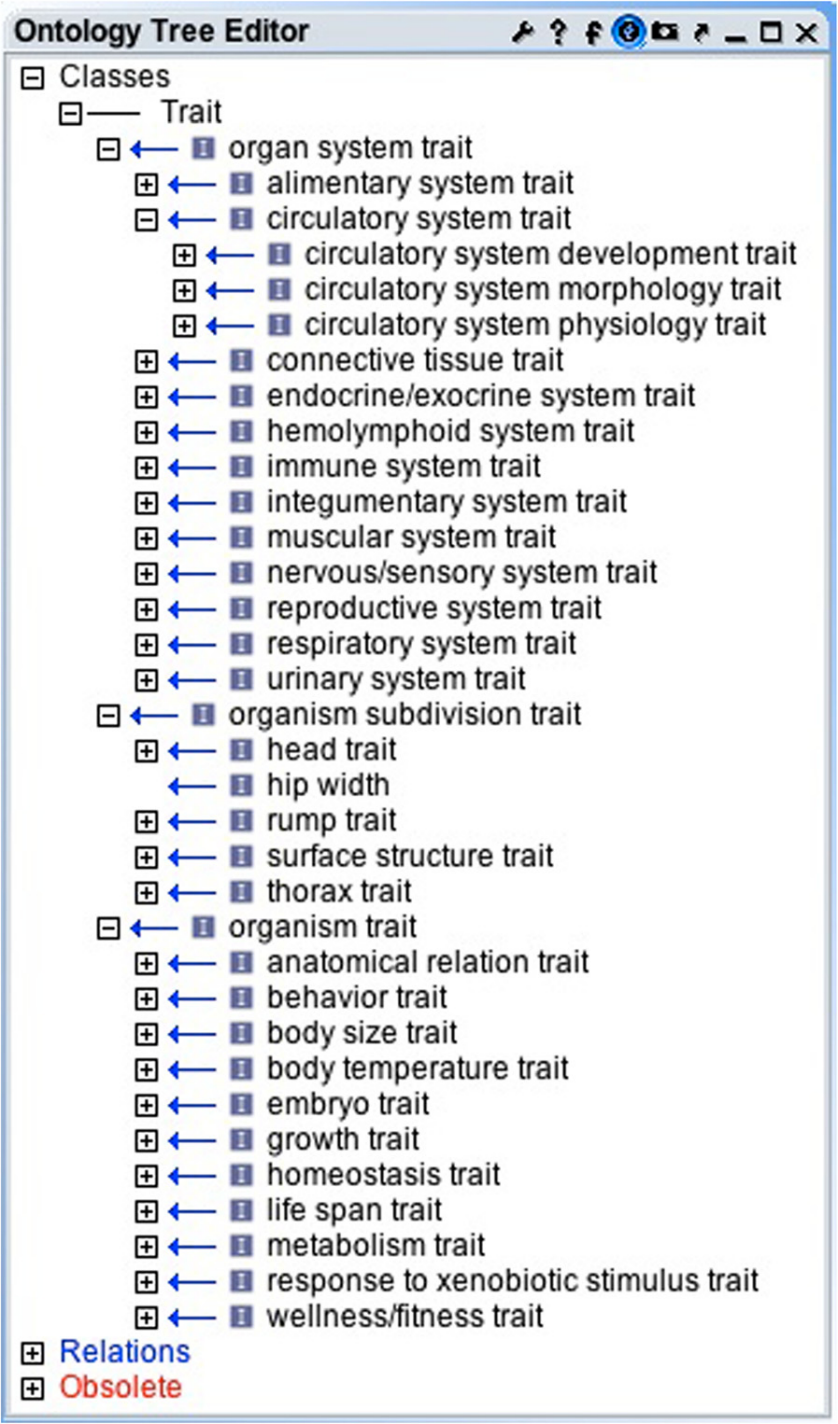
Vertebrate Trait Ontology hierarchy showing upper level terms.

Some reworking of the MP structure was performed to better suit the purposes of the VT. This included addition of new upper level terms, splitting the ontology into three major branches: organ system trait, organism subdivision trait, and organism trait (see Figure 1). Inclusion of traits for non-mammalian species necessitated the generalization of several branches; for example, “skin/coat/nails” from the MP became “integumentary system” and “limbs/digit/tail” became “surface structure.” Various anatomy ontologies, including the Zebrafish Anatomy ontology [26] and the Foundational Model of Anatomy ontology [7], were consulted during this process. These modifications were initiated because of a QTLdb requirement to include traits from chickens, such as beak morphology, wing morphology, and feather morphology traits and egg traits distinct from the female gamete. However, the structure was designed to accommodate all vertebrates.

The VT was developed in the OBO file format using OBO-Edit software, a freely available ontology editor created especially for biological ontologies [27]. The data for each trait term include a unique identifier consisting of the prefix “VT” and a seven-digit number, a definition, a source for the definition (definition dbxref), and pertinent synonyms. To ensure consistency, a list of standard definitions was created for terms that are used frequently throughout the ontology (see Additional file 1). Definitions are often based on the definitions of similar concepts within other ontologies, including PATO. Whenever possible, the term name is species neutral, with species-specific versions consigned to synonyms. For instance, the VT term *longissimus dorsi muscle area* includes the related synonyms *loin eye area* and *ribeye area*, terms commonly used in swine and cattle, respectively. Cross-references to other ontologies, including GO and MP, are provided when highly similar terms are identified. For example, *bitter taste sensitivity trait* is cross referenced to the GO term *sensory perception of bitter taste.* Terms are connected to each other via the is_a relationship, which dictates that more granular, lower-level terms are subtypes of their higher-level parent terms [28]. This relation is transitive, meaning that child terms are not only subtypes of their parent terms, but also of terms further up the hierarchy [29]. The hierarchy takes the form of a directed acyclic graph (DAG), which allows a trait to be a child of multiple parent terms [30].

Ontology development principles set forth by the OBO Foundry, which strives to minimize redundancy and promote interoperability [31,32], have been taken into consideration during creation and development of the Vertebrate Trait Ontology. In adherence to these guidelines, the VT is freely available, versioned, and in a commonly accepted (OBO) format. A unique identifier exists for each term, and nearly all (99.7%) of the terms have textual definitions. Ontology development is collaborative, with cross-references provided to highly similar terms in other ontologies. The VT is continually updated; new traits are added and existing terms are modified to reflect community feedback and to increase accuracy and consistency. The current version contains 3208 terms (v.3.14, http://bioportal.bioontology.org/ontologies/50206?p=terms).

### Utility and discussion

Historically, a combination of QTL name, trait, and subtrait (RGD); trait class, trait type, and trait (QTLdb); a trait class based on an MP term (MPD); or a Mammalian Phenotype term (MGI) was used to define the genetically determined, observed characteristic linked to a genomic region of interest. Although efforts were made by both RGD and QTLdb to standardize this information, the entries were free text, resulting in a diverse array of terms. These included conditions, assay names, disease names, and details of methods used for determining phenotypes, thereby making searching, retrieval, and categorization of the data difficult, if not impossible. Table 1 lists some of the problems with naming conventions that have been corrected by annotation with standardized ontology terms, including VT, CMO, Measurement Method Ontology (MMO), and Experimental Condition Ontology (XCO) [23]. As shown, the original “traits” contained additional information which, though important, does not qualify as legitimate trait data. Likewise, Table 2 demonstrates the wide variety of descriptors that have been distilled down to a single VT assignment (VT:2000000, *arterial blood pressure trait*) for each QTL. The additional data previously found in the subtrait field have now been included in other, more appropriate fields or ontology assignments. Such corrections are currently being made for all rat QTL using these ontologies.

**Table 1:** Problems and their fixes using VT

**Problem**	**Original trait/subtrait or term name**	**Current VT term**	**Current VT ID**
Original QTL “trait” is not a trait:	Hypothalamic-pituitary-adrenal axis/	Blood glucocorticoid amount	VT:0003366
	corticosterone		
	White spotting on belly	Coat/hair pigmentation trait	VT:0010463
Name contains sample information:	Hormone level/	Blood aldosterone amount	VT:0005346
	aldosterone, females		
Name contains experimental condition information:	Blood pressure/	Arterial blood pressure trait	VT:2000000
	salt-depleted		
	Percentage live sperm after thawing	Sperm quantity	VT:0002673
Name contains measurement information:	Blood pressure/	Arterial blood pressure trait	VT:2000000
	pulse pressure		
	Average daily gain	Postnatal growth trait	VT:0001731
Name contains both condition and measurement information:	Post-weaning average daily gain	Postnatal growth trait	VT:0001731
Name contains both method and measurement information:	Blood pressure/	Arterial blood pressure trait	VT:2000000
	direct systolic		
Name contains disease information:	Glucose level/	Blood glucose amount	VT:0000188
	insulin-dependent		
	Tibial dyschondroplasia	Tibia morphology trait	VT:0000558
Same trait described in two different ways:	Gland mass/pancreas	Pancreas mass	VT:0010144
	Pancreas weight/*(none)*	Pancreas mass	VT:0010144

**Table 2:** Standardization of traits

**Trait_name**	**Subtrait_name**
Blood pressure	None
Blood pressure	Arterial
Blood pressure	Diastolic
Blood pressure	Diastolic, daytime
Blood pressure	Direct systolic
Blood pressure	Indirect systolic
Blood pressure	Mean arterial
Blood pressure	Mean arterial pressure
Blood pressure	Mean arterial pressure, stress related changes
Blood pressure	NaCl-loaded systolic blood pressure
Blood pressure	Post nitric oxide system block
Blood pressure	Post renin-angiotensin system block
Blood pressure	Post sympathetic nervous system block
Blood pressure	Pulse pressure
Blood pressure	Response to intrathecal cytisine
Blood pressure	Salt-depleted
Blood pressure	Salt-loaded
Blood pressure	Salt-loaded mean arterial
Blood pressure	Salt-loaded systolic
Blood pressure	Systolic
Blood pressure	Systolic, nighttime

Original RGD trait and subtrait assignments for QTL now annotated with the VT term *arterial blood pressure trait*.

The use of MP terms to drive development of trait classifications by MPD allowed for more standardization but still resulted in inclusion of terms that are not true traits. For example, MPD includes classes for lung tumors and chromosome instability. In addition, use of MP terms to annotate QTL in MGI is problematic. Although the terms are controlled, annotation of QTL to these terms implies that these variants cause abnormality. For example, the C3H allele of the QTL Bnszq2 is annotated to the MP term *decreased compact bone thickness* (MP:0000135). Although this is correct when the C3H strain is compared to the C57BL/6J strain, the transitive nature of the MP implies that this is an abnormal bone morphology annotation, which is not correct. Annotation to the VT term *compact bone thickness* (VT:0000134) is more appropriate.

**Figure 2:**
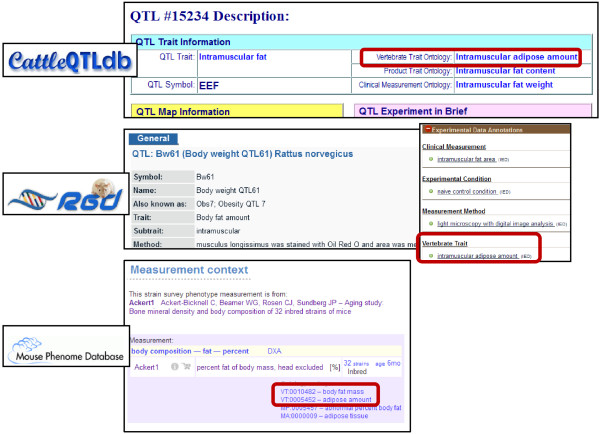
**Database integration of the Vertebrate Trait Ontology.** The Animal QTLdb, RGD, and MPD all annotate QTL with VT terms, facilitating cross-species comparisons. Although the legacy trait and subtrait information are still displayed at the top of the RGD QTL report pages, annotations for VT, CMO, MMO, and XCO are shown in the “Experimental Data Annotations” section of the page, giving users a clear, concise, and standardized list of the trait assessed, the measurement used to assess that trait, the method by which the measurement was made, and the conditions under which the experimental data were obtained.

Currently, the VT is actively being used for annotation of QTL and strain data by QTLdb, RGD, and MPD (see Figure 2). In the QTLdb, existing trait designations have been mapped to applicable terms from the VT and CMO as well as to the livestock Product Trait Ontology (PT), a vocabulary for the measurable or observable characteristics of products produced by or obtained from animals maintained for use or profit [33]. Annotations for at least one of the incorporated ontologies have been added for 94% of the QTL in the database. Annotations to the VT have been made for 66% of the QTL. At RGD, approximately 70% of the rat QTL contain a new “Experimental Data Annotations” section consisting of annotations for VT as well as for CMO, MMO, and XCO. All strain measurements in MPD are now being annotated to the VT. In cases where the measurement value for one or more strains falls outside the normal range, annotations are also made to the MP.

**Figure 3:**
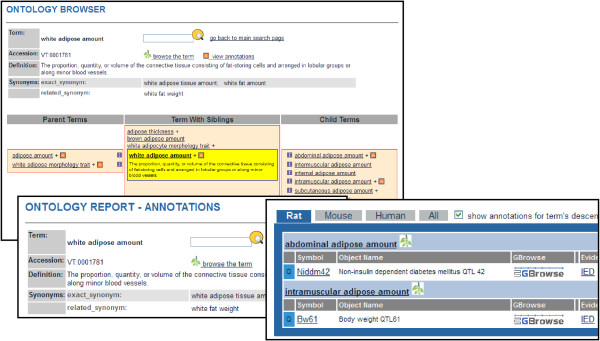
**RGD’s ontology browser and ontology report page.** Both browser and report pages show detailed information about the ontology term and its placement in the ontology structure. The ontology report page displays objects annotated to that page’s term and to any more specific child terms under it in the ontology.

Since the VT terms have been defined, as previously stated, assignment of the appropriate term can be standardized across curators, reducing problems with inter-curator differences in either interpretation or wording. One advantage of expressing these data via ontology annotations is that data can be browsed via the ontology trees. MPD’s “Phenotype strain surveys” page gives users several options for browsing the data, including browsing through the VT ontology tree. Only nodes which link to MPD data are shown, and for each term the number of records annotated to that term and to child term(s) underneath it are displayed. Similarly, because the Vertebrate Trait Ontology has been incorporated into the ontology browser and search tool at RGD, a researcher interested in finding all QTL associated with a particular trait can easily access and display the list. In addition, the structure of the ontology can be leveraged to find not only the QTL associated with a single trait, but also QTL annotated to a term and its more specific child terms, thus expanding the scope of the results without multiple searches. As shown in Figure 3, browsing the VT ontology and viewing the ontology report page for VT:0001781, *white adipose amount*, brings up results for both child terms *abdominal adipose amount* (VT:1000220) and *intramuscular adipose amount* (VT:0010044), so that QTL annotated to both terms may be explored. Similar tools for viewing of QTL by VT terms are currently under development at Animal QTLdb.

**Figure 4:**
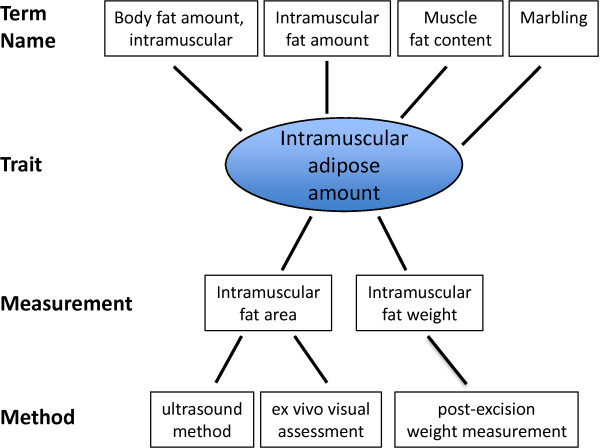
Relationship between term names, trait, measurements, and measurement methods.

RGD is also using the VT ontology to standardize experiment names in the PhenoMiner database [34]. Because multiple measurements and measurement methods can be used to assess a single trait (see Figure 4), using the VT to group such measurements is an obvious solution. In this way, the results for a single trait can be grouped across studies, measurement types, measurement methods, and experimental conditions. Table 3 lists two examples in which a single trait is assessed using multiple clinical measurements.

**Table 3:** A single trait can be assessed via multiple measurements

**Trait assessed: fear/anxiety-related behavior trait, VT:1000241**		
**Measurements:**	Amount of experiment time spent in a discrete space in an experimental apparatus	CMO:0000958
	Number of entries into a discrete space in an experimental apparatus	CMO:0000960
	Percentage of entries into a discrete space in an experimental apparatus	CMO:0000961
	Defecation measurement	CMO:0000997
	Time to first movement outside a discrete space in an experimental apparatus	CMO:0001037
	Number of stretched-attend posture movements	CMO:0001039
	Number of prompted entries into a discrete space in an experimental apparatus	CMO:0001040
	Number of unprompted entries into a discrete space in an experimental apparatus	CMO:0001041
	Number of periods of voluntary immobility	CMO:0001045
**Trait assessed: lymphocyte quantity, VT:0000717**		
**Measurements:**	CD4 cell to CD8 cell ratio	CMO:0000598
	CD4 cell to R73 cell ratio	CMO:0001121
	CD8 cell to R73 cell ratio	CMO:0001122
	R73 cell to total mononuclear cell ratio	CMO:0001120

To demonstrate that these measurements are related, rather than being separate entities, the records are grouped in the PhenoMiner database using the VT term for the trait assessed.

A number of projects involving the VT are currently in early stages. Annotation of mouse QTL with VT terms by MGI is underway and expected to be made public in the future. Also, work is in progress to leverage the structure of VT terms, i.e., the fact that each term consists of both an entity and a quality, in order to decompose them into component terms to improve machine readability.

Finally, we envision that each trait could serve as a single entry point into a wealth of related data. Consider the trait *blood glucose amount*, VT:0000188. Data already linked to this term include rat, cattle, pig, and chicken QTL and mouse strains. In addition, this trait could be linked to related terms in other ontologies such as the CMO, MP, MEDIC Disease Ontology [35], Pathway Ontology (PW; [36]), Chemical Entities of Biological Interest (ChEBI; [9]), and GO Biological Process and Molecular Function. Such mappings would provide further links between the diverse data annotated to them. In this way, a researcher accessing such a trait portal to view information related to blood glucose amount could also access genes, strains, and/or QTL annotated to MP terms such as *increased circulating glucose level* or *abnormal glucose tolerance*; disease terms such as *Diabetes Mellitus* or *Glucose/Galactose Malabsorption*; PW terms related to glucose homeostasis, glucose-related signaling, or anti-diabetic drug pathways; and GO terms ranging from glucose metabolic processes and activities to cellular and organismal responses to glucose (see Additional file 2). Alternatively, researchers could begin with the data already annotated to their terms of interest and explore what other annotations that group of objects is associated with. Such a researcher could start with all QTL associated with *blood glucose amount* and see which CMO, MMO, XCO, MP, and disease terms are also associated with those data objects, thereby getting an overview of the types of experimentation related to that trait and the abnormal phenotypes and diseases demonstrated to be linked to it. Such functionality would give researchers the ability to leverage data of multiple types across multiple species in a single consolidated tool.

## Conclusions

The annotation of genomic data with ontology terms provides unique opportunities for data mining and analysis. Links between data in disparate databases can be identified and explored, a strategy that is particularly useful for cross-species comparisons or in situations involving inconsistent terminology [37,38]. The Vertebrate Trait Ontology provides a common basis for the description of measurable or observable characteristics in multiple vertebrate species. It is already being used, in conjunction with other ontologies, for the annotation of QTL data for rat, cattle, pig, chicken, sheep, and rainbow trout. When multiple ontologies are used to annotate data, more avenues are available for comparison and integration. Since the QTLdb and RGD have already started annotating QTL with VT terms and MPD has linked the VT to strain data, these terms can be used in ontology browsers and searches to extract the annotated data. This provides a starting point for annotating other species with the VT and visualizing all the data at a glance.

### Availability and requirements

This ontology is free and open to all users. It is available for public viewing and download at http://bioportal.bioontology.org/ontologies/50138.

## Abbreviations

CMO: Clinical measurement ontology; DAG: Directed acyclic graph; GO: Gene ontology; INRA: National Institute for Agricultural Research (France); MGI: Mouse genome informatics; MMO: Measurement method ontology; MP: Mammalian phenotype ontology; MPD: Mouse phenome database; NCBO: National center for biomedical ontology; OBO: Open biomedical ontologies; PT: Product trait ontology; PW: Pathway ontology; QTL: Quantitative trait locus/loci; QTLdb: Animal QTL database; RGD: Rat genome database; UMLS: United medical language system; VT: Vertebrate trait ontology; XCO: Experimental condition ontology.

## Competing interests

The authors declare that they have no competing interests.

## Authors’ contributions

CP drafted the manuscript, carried out evaluation of VT structure, crafted term definitions, and utilized the VT for data annotation. SB carried out evaluation of structure, crafted definitions, and helped to draft the manuscript. CS carried out evaluation of structure, crafted definitions, and helped to draft the manuscript. ZH participated in planning of the project and facilitated utilization of the VT for data annotation. DM carried out evaluation of structure and crafted definitions. RN utilized the VT for data annotation and helped to draft the manuscript. JS utilized the VT for data annotation, crafted definitions, and helped to draft the manuscript. MS conceived of the study, participated in design and coordination, and helped to draft the manuscript. JE participated in initial design and coordination. JR conceived of the study, participated in design and coordination, and helped to draft the manuscript. All authors read and approved the final manuscript.

## Supplementary Material

Additional file 1 **Commonly used definitions.** This file includes standard core definitions for terms used frequently throughout the ontology. Click here for file

Additional file 2 **Mockup of hypothetical cross-species data portal.** This figure provides an example of a putative “trait portal,” which would allow users to view large amounts of related data via a single entry point. Click here for file
