# Supplementary material for: The Vertebrate Trait Ontology: a controlled vocabulary for the annotation of trait data across species
Source: J Biomed Semantics. 2013 Aug 9;4:13. doi: 10.1186/2041-1480-4-13 (PMC3851175; doi:10.1186/2041-1480-4-13)
Supplement: Additional file 2 — Mockup of hypothetical cross-species data portal. This figure provides an example of a putative “trait portal,” which would allow users to view large amounts of related data via a single entry point. [file 2041-1480-4-13-S2.pptx]

## Slide 1
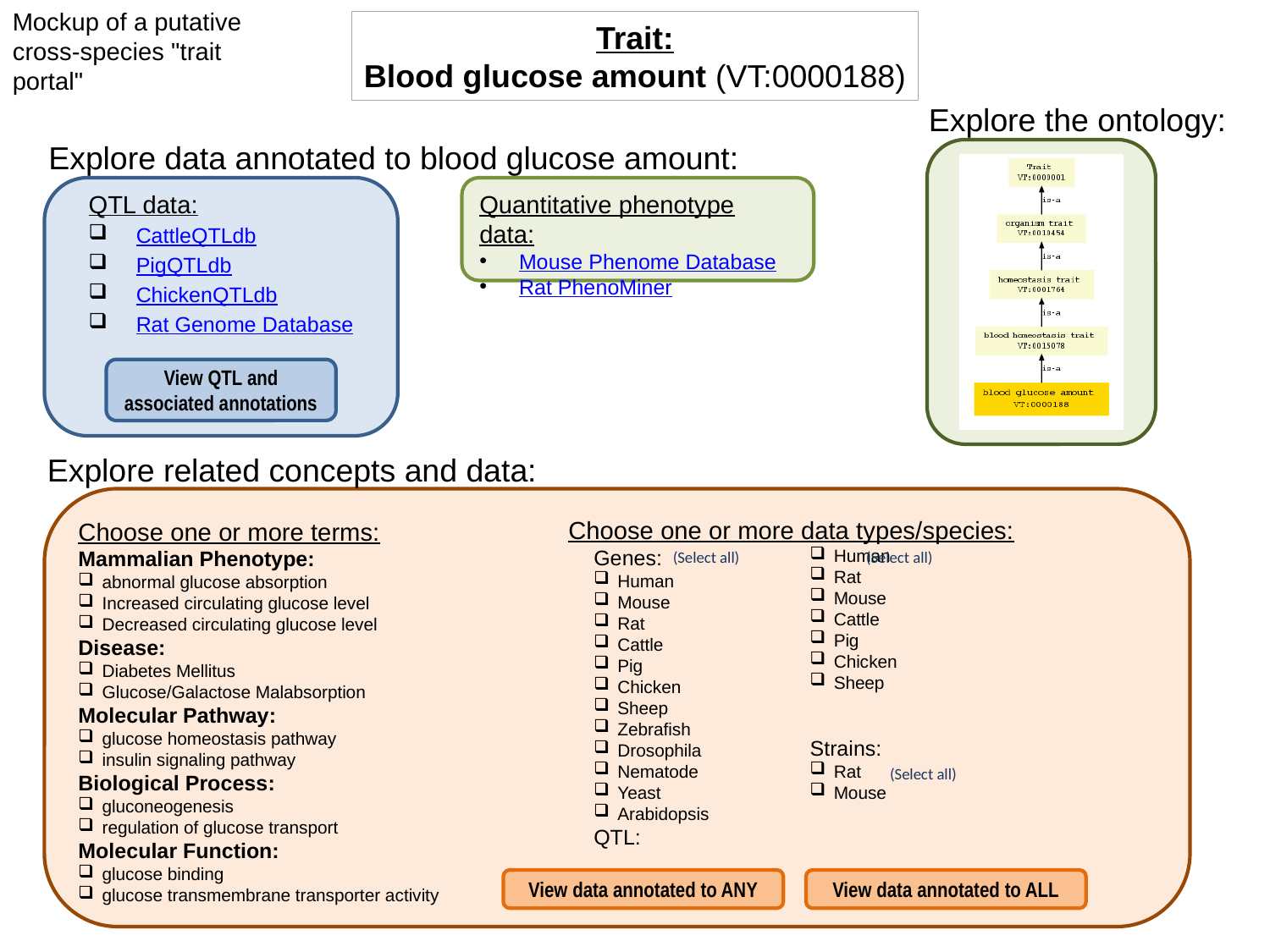

Mockup of a putative cross-species "trait portal"
Trait:
Blood glucose amount (VT:0000188)
Explore the ontology:
Explore data annotated to blood glucose amount:
Quantitative phenotype data:
Mouse Phenome Database
Rat PhenoMiner
QTL data:
CattleQTLdb
PigQTLdb
ChickenQTLdb
Rat Genome Database
View QTL and associated annotations
Explore related concepts and data:
Choose one or more terms:
Mammalian Phenotype:
abnormal glucose absorption
Increased circulating glucose level
Decreased circulating glucose level
Disease:
Diabetes Mellitus
Glucose/Galactose Malabsorption
Molecular Pathway:
glucose homeostasis pathway
insulin signaling pathway
Biological Process:
gluconeogenesis
regulation of glucose transport
Molecular Function:
glucose binding
glucose transmembrane transporter activity
Choose one or more data types/species:
Genes:
Human
Mouse
Rat
Cattle
Pig
Chicken
Sheep
Zebrafish
Drosophila
Nematode
Yeast
Arabidopsis
QTL:
Human
Rat
Mouse
Cattle
Pig
Chicken
Sheep
Strains:
Rat
Mouse
(Select all)
(Select all)
(Select all)
View data annotated to ANY
View data annotated to ALL
